# Supplementary material for: FERN – a Java framework for stochastic simulation and evaluation of reaction networks
Source: BMC Bioinformatics. 2008 Aug 29;9:356. doi: 10.1186/1471-2105-9-356 (PMC2553347; doi:10.1186/1471-2105-9-356)
Supplement: Additional file 1 — FERN distribution, Version 1.3. This archive contains the FERN source code and binaries as well as documentation and example models in FernML and SBML. [file 1471-2105-9-356-S1.zip › fern/doc/javadoc/fern/simulation/observer/class-use/Observer.html]

Uses of Class fern.simulation.observer.Observer


---


|  |  |  |
| --- | --- | --- |
| **Overview** \| **Package** \| **Class** \| **Use** \| **Tree** \| **Deprecated** \| **Index** \| **Help** | |  |
| PREV   NEXT | **FRAMES**    **NO FRAMES**     **All Classes** |  |


---


## **Uses of Class fern.simulation.observer.Observer**

| Packages that use Observer | |
| --- | --- |
| **fern.cytoscape** | Provides the classes for the cytoscape plugin. |
| **fern.example** | Contains examples and demonstrations of the framework. |
| **fern.network.sbml** | Provides the classes for parsing and using sbml based networks. |
| **fern.simulation** | Provides algorithms for simulating reaction network as well as methods for observer certain aspects of a simulation. |
| **fern.simulation.observer** | Provides classes to observe certain aspects of simulations. |

Uses of Observer in fern.cytoscape

| Subclasses of Observer in fern.cytoscape | |
| --- | --- |
| `class` | `CytoscapeColorChangeObserver` |

Uses of Observer in fern.example

| Subclasses of Observer in fern.example | |
| --- | --- |
| `class` | `CellGrowthObserver`             Does not observe anything, but it controls the reaction networks cell. |

Uses of Observer in fern.network.sbml

| Subclasses of Observer in fern.network.sbml | |
| --- | --- |
| `class` | `SBMLEventHandlerObserver`             Observer which handles an event of a sbml model. |

Uses of Observer in fern.simulation

| Methods in fern.simulation that return Observer | |
| --- | --- |
| `Observer` | `Simulator.addObserver(Observer observer)`             Adds an observer to the list of observers. |

| Methods in fern.simulation with parameters of type Observer | |
| --- | --- |
| `Observer` | `Simulator.addObserver(Observer observer)`             Adds an observer to the list of observers. |
| `void` | `Simulator.registerNewTheta(Observer obs, double theta)`             Is called by `setTheta(double)`. |

Uses of Observer in fern.simulation.observer

| Subclasses of Observer in fern.simulation.observer | |
| --- | --- |
| `class` | `AmountAtMomentObserver`             Observes the amount of some molecule species at a certain moment. |
| `class` | `AmountIntervalObserver`             Observes amounts of molecule species repeatedly after certain intervals. |
| `class` | `FireTypeObserver`             Observes the different types of firings. |
| `class` | `InstantOutputObserver`             Simply prints out every event that the simulator reports to the observer. |
| `class` | `IntervalObserver`             Base class for observing certain aspects repeatedly after a given interval. |
| `class` | `LeapObserver`             Observes the amount of given molecule species at each `step`. |
| `class` | `MultiAmountIntervalObserver`             Observes amounts of molecule species repeatedly after certain intervals. |
| `class` | `RandomNumberGeneratorCallObserver`             Observes the calls to the random number generator and count the number of drawn random number of different distributions. |
| `class` | `ReactionIntervalObserver`             Observes number of firings of reactions repeatedly after certain intervals. |
| `class` | `TriggerObserver` |

---


|  |  |  |
| --- | --- | --- |
| **Overview** \| **Package** \| **Class** \| **Use** \| **Tree** \| **Deprecated** \| **Index** \| **Help** | |  |
| PREV   NEXT | **FRAMES**    **NO FRAMES**     **All Classes** |  |


---
